# Acute retinal detachment after Nd:YAG treatment for vitreous floaters and postertior capsule opacification: a case report

**DOI:** 10.1186/s12886-020-01428-7

**Published:** 2020-04-19

**Authors:** Xiuduo Liu, Qian Wang, Jie Zhao

**Affiliations:** Yangpu district shidong hospital, Shanghai, China

**Keywords:** Nd:YAG vitreolysis;vitreous floaters, Capsulotomy, Retinal detachment

## Abstract

**Background:**

Modern laser surgery uses Nd:YAG laser capsulotomy for posterior capsule opacification (PCO) and Nd:YAG laser vitreolysis for symptomatic vitreous floaters (VF). We report a case of acute retinal detachment seven days after Nd:YAG laser capsulotomy combined with Nd:YAG laser vitreolysis and analyze the cause of this complication.

**Case presentation:**

A 58-year-old myopic woman complained of decreased visual acuity and symptomatic floaters with her left eye for 3 months. We found she had significant PCO and VF in the posterior vitreous. She underwent neodymium-doped yttrium aluminum (Nd:YAG) laser vitreolysis immediately after Nd:YAG capsulotomy. After 7 days, she complained of rapid vision decline and dark shadows in her treated eye. We found she had a acute severe rhegmentogenous retinal detachment (RD) involving the macula. Then she underwent vitrectomy, retinal reattchment and silicone oil tamponade surgery immediately. Six months later, silicone oil was removed and the best corrected visual acuity (BCVA) of her left eye gradually improved to 10/20 and maintained during a 1-year follow-up period.

**Conclusion:**

As myopic patients are at risk of developing retinal detachment, Nd:YAG vitreolysis and capsulotomy should be performed with caution. The laser energy should be as low as possible and careful focus is necessary to reduce interference to the retina.

## Background

Symptomatic floater is the common disease of the ophthalmological clinics. Because it may interfere with the patients’ quality of vision, many patients are eager to remove their floaters. Webb [1] reported that floaters create anxiety in 76% of patients and significantly decrease the quality of life in 33% of them. In a study by Wagle et al. [2] revealed that patients with symptomatic floaters would rather sacrifice 1.1 year for every 10 years of their remaining life in order to eliminate the discomfort caused by their floaters. Currently, the main treatment methods for symptomatic floaters are ND:YAG laser vitreolysis and vitrectomy. Compared with vitrectomy, Nd:YAG laser vitreolysis is much more simple, safe and economical. Complication of Nd:YAG laser vitreolysis have been reported such as cataract and glaucoma [3, 4]. In this case we reported a patient with PCO and VF who developed severe rhementogenous retinal detachment 7 days after ND:YAG laser capsulotomy and vitreolysis.

## Case presentation

**Fig. 1:**
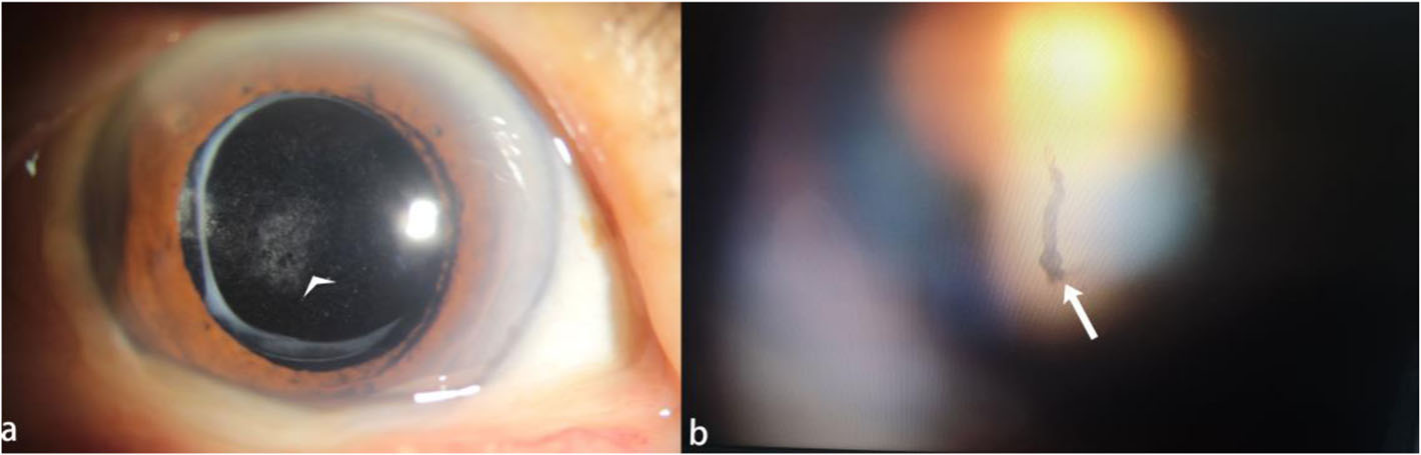
**a** Elsching pearl like PCO (arrowhead). **b** vitreous floaters in the posterior segment of the vitreous cavity (arrow)

**Fig. 2:**
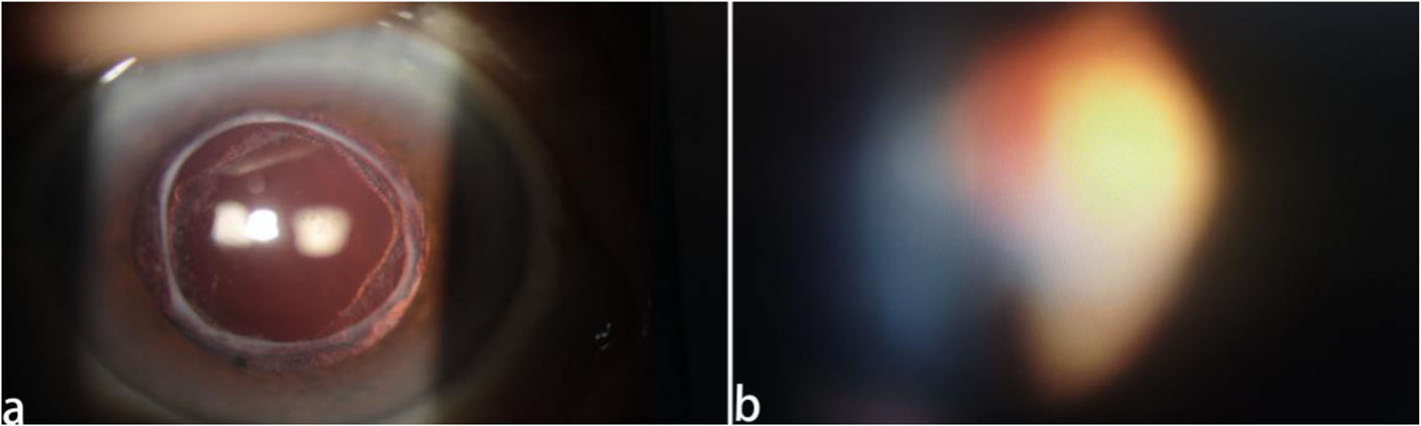
**a** cleared posterior capsular opacity by Nd:YAG laser capsulotomy. **b** cleared vitreous floaters in the posterior segment of the vitreous cavity by Nd:YAG laser vitreolysis

**Fig. 3:**
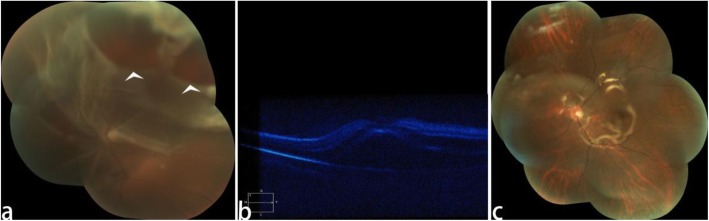
the retina 7 days after Nd:YAG laser treatment **(a)** color fundus photograph of the left eye demonstrating the retina of upper and nasal part detachment with two large holes in the detached upper retina. **b** the optical coherence tomography show the retinal detachment involved the macular fovea. **c** postoperative color fundus photograph of vitrectomy, retinal reattchment and silicone oil tamponade surgery

A 58-year-old myopic woman visited our clinic, complaining of blurred vision and symptomatic floaters in her left eye for 3 months. Ophthalmologic examination revealed significant Elsching pearl like PCO in her left eye and VF like a thick string in the posterior segment of the vitreous cavity(Fig. 1a). The BCVA of her left eye was 8/20. She had no history of any systemic disease and drug intake. Two years ago she had undergone phacoempulsification and IOL implantation in her left eye, preoperatively she had − 6.50DS myopia with her left eye and the axial length of her left eye was 26.81 mm. We performed vitreous and retina examination on her left eye by slit-lamp biomicroscopy with Goldman’s three mirror contact lens to rule out peripheral retinal tears and degeneration. By Goldmann three-mirror lens, we clearly saw the partial posterior vitreous detachment (PVD) at posterior fundus and the VF was approximately 4-5 mm in front of the optic disc(Fig. 1b). The patient was eager to improve the visual acuity and remove the floaters in her left eye. At that time, we considered that the patient’s residence was far away from our clinic. In order to reduce the number of visits of the patient, we decided to complete Nd:YAG laser capsulotomy and Nd:YAG laser vitreolysis in one time. After signing the informed consent, Nd:YAG capsulotomy was performed first, followed by Nd:YAG laser vitreolysis immediately. In order to decrease the symptoms of floaters after Nd:YAG capsulotomy, we used the cross incision method for Nd:YAG capsulotomy. The diameter of Nd:YAG capsulotomy was about 6 mm(Fig. 2a), the power was set from 1.6 to 3.0 mJ, and the total power was 34 mJ. Then we swithed the offset to the anterior to avoid the damage of the retina and optic disc behind the VF. We used the professional vitreous laser lens (the type: KARICKHOFF25mm OFF-AXIS) for vitreolysis. During the vitreolysis the energy ranged from 3.0–4.5 mJ for a value that fully vaporized the VF(Fig. 2b) and the total power of vitreolysis was 130 mJ. In the process of Nd:YAG laser vitreolysis we found the fiber around the floaters were shaking violently. The whole timing of the two laser treatment was about 15 min. After the laser treatment, the patient immediately complained of a prominent darking and flashing sensation with her treated eye, but the patient’s complaint did not catch our attention and we did not have a timely retinal examination on her. Seven days later, she came back to our clinic complaining of rapid vision loss in her left eye and dark shadows in her lower visual field. We found her BCVA of the left eye was down to finger count at 2 ft. We found retinal detachment (RD) at the upper and nasal retina and involved the macular fovea, and two large holes in the upper retina about 4DD in diameter (Fig. 3). She immediately underwent a vitrectomy, retinal reattchment and silicone oil tamponade surgery for her left eye. Six months later silcone oil removal was arranged on her, and the BCVA in her left eye gradually improved to 10/20 and maintained during a 1-year follow-up period.

## Discussion and conclusion

In this case, the patient was treated with Nd:YAG laser capsulotomy and vireolysis therapy for PCO and VF in one procedure. Powell et al. [5] reported that the incidence of RD following Nd:YAG laser capsulotomy was 0.82%,with a mean time of 13.5 months between capsulotomy and RD. A study by Wesolosky et al. [6] reported the cumulative risk of RD at 3, 6, 9, and 12 months after Nd:YAG laser capsulotomy was 0.6,0.96,1.19 and 1.39%,and the rates of RD varied significantly between age categories. In a study by Ranta P et al. [7] reported that by 5 years, the overall cumulative of proportion of RD in the 341 patients was 2.0%, and the axial lenth had strongest association with RD after Nd:YAG laser capsulotomy. The axial lenth of our case was 26.81 mm, but RD occurred as soon as 7 days after Nd:YAG laser capsulotomy has been rarely reported. A recent review [8] reported that Nd:YAG capsulotomy is not associated with RD, thus, it is more likely that RD developed following Nd:YAG vitreolysis rather than posterior capsulotomy. Moreover, the total energy delivered after capsulotomy was 34 mJ, while 130 mJ for vitreolysis. There was no abnormality during Nd:YAG capsulotomy. However, during Nd:YAG vitreolysis, the fiber cables in the posterior segment of the vitreous were found to shake violently, this may be related to higher energy. After vitreolysis the patient immediately complained of a flash sensation. One possible explanation is that due to the 3 DD separation of floater from optic disc, the laser induced rapid completion of PVD and hence the horseshoe tear and the further RD forms. The photodisruption effect of the Nd:YAG laser seems also affects the vitreous fibers around the VF, which could cause traction to the peripheral retina then the retinal tear forms and the higher energy of vitreolysis maybe the another reason of acute RD. There have been few studies reporting the complications following treatment with Nd:YAG vitreolysis. Cowan et al. [3] reported two cases of open angle glaucoma 7 days and 8 months after YAG laser vitreolysis with >40 mmHg of interocular pressure. In addition, some studies reported cataracts [4, 9]; posterior capsule defects requiring cataract surgery; retinal tear; retinal detachment [10]; retinal hemorrhages; scotomas; increased number of floaters [11]. Acute RD as severe as this case has not been reported. The laser energy should be as low as possible and it must be focused on the PCO and vitreous floaters as precisely as possible to reduce interference with retina. It is suggested that Nd:YAG capsulotomy maybe performed first on PCO and then Nd:YAG vitreolysis maybe performed after a safe interval and a thorough retinal examination. It should be alert to the possibility of RD when patients complain of prominent darking and flashing sensation flash sensation after Nd: YAG vitreolysis or capsulotomy. After this case, we did not have any RD complications with Nd: YAG laser therapy in similar patients.

## Data Availability

The datasets used and analysed during the current study are available from the corresponding author on reasonable request.

## Abbreviations

PCO: Posterior capsule opacification;
VF: Vitreous floaters
Nd:YAG: Neodymium-doped yttrium aluminum
BCVA: Best corrected visual acuity
Nd:YAG: Neodyminum-doped yttrium aluminum garnet
RD: Retinal detachment
PVD: Posterior vitreous detachment
